# Region-level epimutation rates in *Arabidopsis thaliana*

**DOI:** 10.1038/s41437-021-00441-w

**Published:** 2021-05-08

**Authors:** Johanna Denkena, Frank Johannes, Maria Colomé-Tatché

**Affiliations:** 1 Institute of Computational Biology, Helmholtz Zentrum München, Neuherberg, Germany; 2 Department of Molecular Life Sciences, Hans Eisenmann-Zentrum for Agricultural Sciences, Technical University Munich, Freising, Germany; 3 Biomedical Center, Physiological Chemistry, Faculty of Medicine, LMU Munich, Planegg-Martinsried, Germany

**Keywords:** Epigenomics, Natural variation in plants

## Abstract

Failure to maintain DNA methylation patterns during plant development can occasionally give rise to so-called “spontaneous epimutations”. These stochastic methylation changes are sometimes heritable across generations and thus accumulate in plant genomes over time. Recent evidence indicates that spontaneous epimutations have a major role in shaping patterns of methylation diversity in plant populations. Using single CG dinucleotides as units of analysis, previous work has shown that the epimutation rate is several orders of magnitude higher than the genetic mutation rate. While these large rate differences have obvious implications for understanding genome-methylome co-evolution, the functional relevance of single CG methylation changes remains questionable. In contrast to single CG, solid experimental evidence has linked methylation gains and losses in larger genomic regions with transcriptional variation and heritable phenotypic effects. Here we show that such region-level changes arise stochastically at about the same rate as those at individual CG sites, are only marginal dependent on region size and cytosine density, but strongly dependent on chromosomal location. We also find consistent evidence that region-level epimutations are not restricted to CG contexts but also frequently occur in non-CG regions at the genome-wide scale. Taken together, our results support the view that many differentially methylated regions (DMRs) in natural populations originate from epimutation events and may not be effectively tagged by proximal SNPs. This possibility reinforces the need for epigenome-wide association studies (EWAS) in plants as a way to identify the epigenetic basis of complex traits.

## Introduction

Cytosine methylation is an epigenetic modification with important roles in the silencing of transposable elements (TEs), the formation of heterochromatin, and the regulation of some genes (Kawakatsu et al. 2016). While cytosine methylation in mammals occurs almost exclusively in CG context (CpG), the methylation of cytosines in plants is also abundant in the CHG and CHH contexts (H = C, T or A) (Law and Jacobsen 2010). In plants, methylation of cytosines in each of the three contexts is maintained by different well-characterized pathways, which are broadly conserved across taxa, suggesting that proper DNA methylation is subject to strong evolutionary constraints (Law and Jacobsen 2010). Nonetheless, stochastic losses and gains of methylation can arise in plant genomes independently of genetic mutations (Becker et al. 2011; Schmitz et al. 2011), probably as a byproduct of imperfect maintenance fidelity across cell divisions (Hofmeister et al. 2020; Shahryary et al. 2020). Once acquired, these so-called “spontaneous epimutations” are heritable over many generations and have the potential to affect the transcriptional output of nearby genes (Schmitz et al. 2013a). Recent analyses of natural populations of *Arabidopsis* and maize have provided strong indications that the accumulation of spontaneous epimutations has a major role in shaping methylation diversity patterns over evolutionary time (Vidalis et al. 2016; Xu et al. 2020), although its role in adaptive processes remains unclear (Johannes and Schmitz 2019; Seymour and Becker 2017).

Quantitative insights into the formation and trans-generational inheritance of spontaneous epimutations have come from careful studies of mutation accumulation (MA) lines. MA lines are populations of plants that are derived from a single founder and propagated over multiple generations in a stable environment (Becker et al. 2011; Schmitz et al. 2011; Shaw et al. 2000). Using MA lines of the model plant *Arabidopsis thaliana*, van der Graaf et al. (2015) estimated the rate at which single cytosines in CG context gain methylation (gain rate *α*) at 2.56 · 10^−4^ per generation per haploid methylome and the loss of methylation (loss rate *β*) at 6.30 · 10^−4^ per generation per haploid methylome. These rates are about 5 orders of magnitude higher than the genetic mutation rate of 6.95 · 10^−9^ calculated for *A. thaliana* by Weng et al. (2019). These large rate differences predict that methylome diversity arises much more rapidly than genomic diversity in natural populations, and that epigenetic variation becomes uncoupled from genetic variation over evolutionary time scales (van der Graaf et al. 2015). One important practical implication of this is that segregating epimutations and their potential functional consequences are not effectively tagged by proximal SNPs in genome-wide association studies (Johannes et al. 2008).

The relevance of these insights can be questioned on the grounds that there is currently no evidence that methylation status changes at single CGs have any functional consequences in plants. By contrast, gains and losses of methylation over larger regions have been repeatedly linked to heritable phenotypic variation in a number of plant species (Cubas et al. 1999; Gallusci et al. 2016; Ong-Abdullah et al. 2015). Several studies have reported that such regions-level changes (ranging from 50 bp to 1 kb in length) are rare, but do occur in *A. thaliana* MA lines at about the same frequencies as genetic mutations (Becker et al. 2011; Ganguly et al. 2017; Hofmeister et al. 2017; Jiang et al. 2014; Schmitz et al. 2011). Some subsequent publications interpreted this to mean that the epimutation rate for regions is comparable with the genetic mutation rate. However, the number of “epimutable” sites in the genome (i.e., regions containing clusters of cytosines) is far smaller than the number of “genetically mutable” sites (i.e., all nucleotides), which would imply that region-level epimutations rates are actually much higher than the genetic mutation rate. Yet, this hypothesis has never been explored formally.

Here, we provide the first estimates of regions-level epimutation rates in the model plant *A. thaliana*. Our results show that the rate and spectrum of region-level epimutations are similar to that observed for single CG epimutations. To gain insights into the predictors of spontaneous epimutations, we study the relationship between genome annotation (like genes or TEs) and epimutation rates. We also investigate epimutation rate levels in distinct chromosomal regions ((peri-)centromeres and chromosome arms) as well as regions of different sizes and densities. Finally, we compare the found epimutation rates to mutation rates in the same genomic regions. Our results have major implications for understanding how linkage disequilibrium (LD) between genetic variants and differentially methylated regions (DMRs) evolves over time and reinforces the need to carry out methylation-based epigenome-wide association studies (EWAS) in plants.

## Materials and methods

### MA lines

**Fig. 1:**
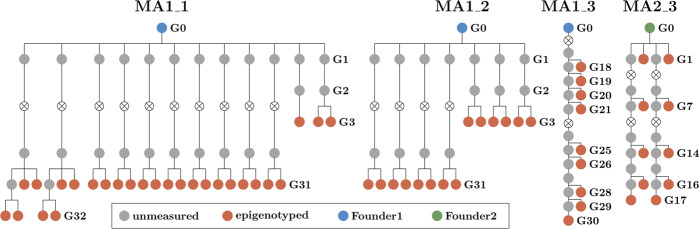
Pedigrees for the mutation accumulation lines MA1_1, MA1_2, MA1_3, and MA2_3. The unmeasured plants are denoted in grey, while plants that were sequenced are marked red. The generations (“Gn") of the plants are added next to the pedigrees. The blue and green color of the founder plants show that MA1_1, MA1_2 and MA1_3 have a different founder form MA2_3.

To estimate epimutation rates, we used whole-genome-bisulfite-sequencing (WGBS) data from four previously published *A. thaliana* MA data sets (Fig. 1). The pedigrees MA1_1, MA1_2, and MA1_3 were generated from the same common Col-0 plant founder MA1, while the pedigree MA2_3 was generated from a different Col-0 founder. All lines were propagated by single seed descent under stable conditions (Shaw et al. 2000), and one sibling plant was used for propagating the line while another sibling plant was used for sequencing. MA1_1 has been described in Becker et al. (2011). It consists of 12 branches, from which individuals had been sequenced in generations three, 31, and 32 (Fig. 1). MA1_2 has been published by Schmitz et al. (2011) and consists of a pedigree with seven branches with individuals measured in generations three and 31 (Fig. 1). MA1_3 and MA2_3 were previously published in van der Graaf et al. (2015). MA1_3 consists of only one branch sequenced at nine different generations (Fig. 1), while MA2_3 has two branches sequenced at five different generations (Fig. 1).

### Constructing regions

**Fig. 2:**
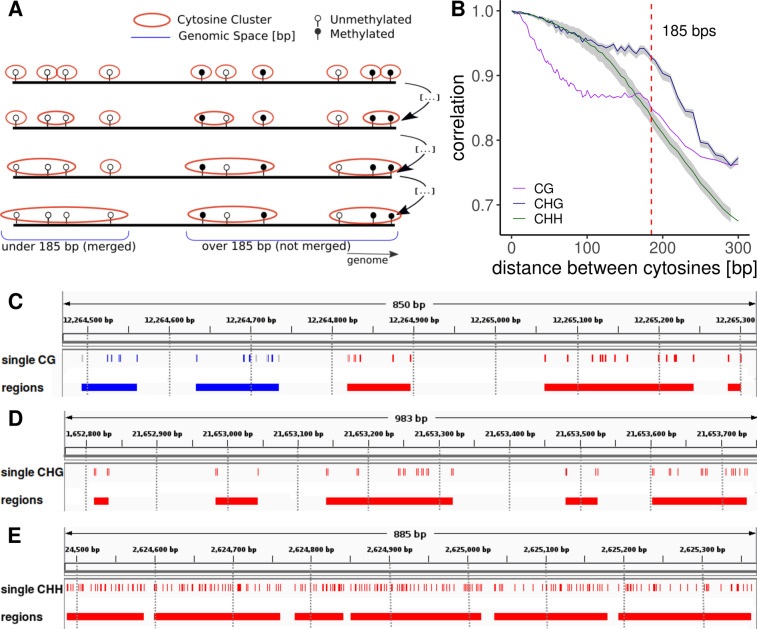
Construction of regions. **A** Regions are constructed by iteratively concatenating cytosines (and later clusters of cytosines) if they are less than 185 bp apart and their combined regions size does not exceed 185 bps (blue line on the left). Clusters further apart than 185 bp are kept separate (blue line on the right). The process starts with the cytosines that are closest together and iterates through increasing distances. **B** Autocorrelation of cytosines per context for MA line MA1_3. For the autocorrelation of the other MA lines see Figs. S1 and S2. The gray shading represents the variation caused by the different samples in the MA line pedigrees. The variation in CG context is included in the plot, but it is smaller than the line width and it is not visible. **C** Example snapshot from the integrative genome viewer (Robinson et al. 2011) showing a stretch of chromosome 1 from MA1_3. The top track shows the single CGs and the bottom track shows the regions. For both tracks, regions/cytosines are either Methylated (blue), unmethylated (red), or insufficiently covered (gray). For comparison, 100 bp bins are marked with vertical lines, showing how partitioning the genome with an arbitrary window size does not necessarily group close cytosines together. **D** Example snapshot of chromosome 1 from MA1_3 showing single CHGs and CHG regions. **E** Example snapshot of chromosome 1 from MA1_3 showing single CHHs and CHH regions.

In order to estimate region-level epimutation rates, we first defined regions or “clusters of cytosines” based on genomic sequence information, separately for each context. Inspired by approaches used in the animal field for identifying CpG islands, our method starts by defining every pair of cytosines that is a minimal genomic distance apart as seed regions (Fig. 2A). In the case of CG, the minimal distance is 0 as every cytosine on the forward strand is complemented by a CG on the backward strand, and there is no nucleotide between the two CGs. For CHG, the minimal distance is 1 and for CHH, because of the lack of symmetry, a seed is formed by every individual CHH on each strand. Then every two seeds with a distance of [minimal distance + 1] between each other are merged. This process is repeated iteratively with increasing distances until either (i) the nearest neighboring region is more than 185 bp apart, or (ii) the combined region length is higher than 185 bp (Fig. 2A). This way every C in the genome is assigned to a CG, CHG, or CHH region, depending on its context.

The 185 bp cutoff was based on the decay of the methylation autocorrelation in the genome, which we calculated between neighboring cytosines per context (CG, CHG, or CHH). As expected, the autocorrelation was higher for cytosines at shorter distances compared to cytosines located further away from each other. For contexts CG and CHG, the autocorrelation slowly decreased until 185 bp, followed by an abrupt decline (Fig. 2B), probably due to the nucleosomic organization of the genome. Therefore we chose 185 bp as the cutoff for the constructed regions. Figure S3 shows how different cutoffs for the construction of regions determine region characteristics. For comparison, we also created regions by binning the genome at 100 bp, as is commonly done by DMR calling software (Fig. 2C).

### Methylation divergence

After having set up the regions on the basis of genomic information, methylated and unmethylated read counts were summed up per region. To circumvent using regions with poor coverage, only regions with on average more than three reads per cytosine (total number of reads in the region/number of cytosines in the region) in all individuals of a pedigree were used for further analysis. We called methylation status in regions using a function from the package METHimpute, an algorithm based on a hidden Markov model (HMM) (Taudt et al. 2018) that takes the number of methylated and unmethylated reads as input to call methylation status. Utilizing the function call Methylation, each region was assigned to one of the three methylation status calls: unmethylated (U), methylated (M), or intermediate (I). Methylation status calls were measured by the maximum posterior probability of the HMM. To avoid methylation calls of poor quality only regions with a maximum posterior probability of at least 0.99 in all individuals of a pedigree were used for further analysis. For every pair of individuals in a pedigree, we calculated the methylation divergence, with respect to the generation time Δ*t*, which is counted as the independent number of selfing events from their most recent common founder (Fig. S5). Following van der Graaf et al. (2015), methylation divergence between two plants *i* and *j*, *d*_*ij*_, was calculated assuming that for region *n* two individual plants have a divergence of 1 if the tuple of their methylation states, (*m*_*i*,n_, *m*_*j*,n_), is discordant ((*M*, *U*) or (*U*, *M*)), of 0.5 if their methylation states are intermediate in one of the two individuals ((*M*, *I*), (*I*, *M*), (*U*, *I*) or (*I*, *U*)), and of 0 if the two individuals have the same methylation status ((*U*, *U*), (*I*, *I*), (*M*, *M*)). The genome-wide divergence between two individuals *i* and *j* in a pedigree is then defined as:

1 $$D_{ij} = \frac{1}{N}\mathop {\sum }\limits_{n = 1}^N d_{ij,n}$$

where

$$d_{ij,n} = \left\{ {\begin{array}{*{20}{c}} {1,if\,m_{i,n},m_{j,n} \in \{ (M,U),(U,M)\} } \\ {0.5,if\,m_{i,n},m_{j,n} \in \{ (M,I),(I,M),(U,I),(I,U)\} } \\ {0,if\,m_{i,n} = m_{j,n}} \end{array}} \right.$$

and *N* as the total number of regions.

### Epimutation rate estimation

Region-based estimates of the methylation gain rate *α* and the loss rate *β* were obtained with the R-package AlphaBeta (Shahryary et al. 2020). The AlphaBeta package assumes that the genome-wide methylation levels are at equilibrium. Since the *α* and *β* rates can only act on their substrate (i.e., *α* can only produce a methylation gain on Cs, while *β* can only produce a methylation loss on mCs), the relationship between the *α* and *β* rate determines equilibrium methylation levels.

We fitted four competing models: ABneutral, ABmm, ABuu, ABnull to the divergence data of each pedigree. Model ABneutral assumes that spontaneous methylation gains and losses accumulate neutrally across generations, ABmm assumes that the accumulation is partly shaped by selection against spontaneous methylation losses, ABuu assumes that the accumulation is partly shaped by selection against spontaneous methylation gains, and ABnull is the null model of no accumulation. Formal model comparisons were carried out as described by Shahryary et al. (2020).

### Annotation specific estimates

We considered methylation accumulation in different genomic regions to investigate which factors had an influence on the epimutation rate estimates. First, regions were categorized by their size and cytosine density. We considered duplets, consisting of two CGs on opposite strands, and longer regions, that we termed multiplets. The longer regions were categorized into high and low cytosine density (above and below the median density of 0.105), as well as long and short regions (splitting them into regions that were shorter /longer than the median size of 104).

Next, we split the regions based on their overlap (≥40% overlap) with genes, TEs, 5′-UTRs, and 3′-UTRs from The Arabidopsis Information Resource (2018), release TAIR10. Promoters were defined as 1.5 kb upstream of each TSS and the genomic space not covered by any of these annotations was defined as intergenic. Genes were further subdivided into gene body methylated genes (gbM genes: mCG enrichment at the gene body, but depletion at Transcription Start and Termination Sites) and non-gbM genes (Bewick et al. 2016). gbM classification was obtained from Bewick et al. (2016), who identified 17% of all genes as gbM in the Col-0 background. Because of overlapping annotations, some regions corresponded to more than one annotation. Finally, we tested differences in epimutation rates between centromeres, pericentromeres, and chromosome arms. We used the coordinates from Weng et al. (2019), which were adapted from Ossowski et al. (2010) by converting them from TAIR8 to TAIR10.

## Results

### Segmentation

**Fig. 3:**
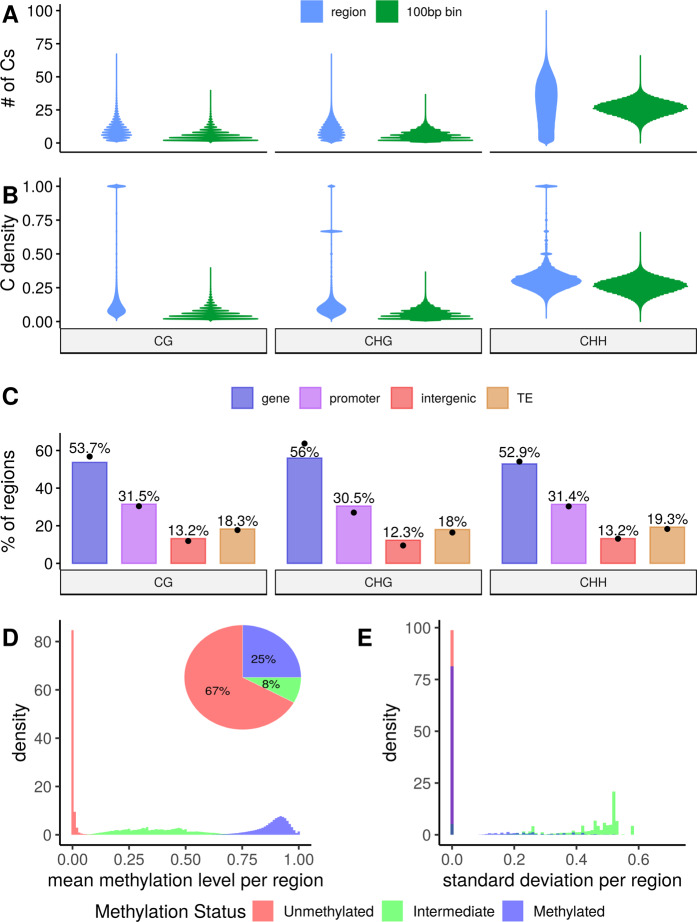
Characteristics of methylation regions. **A** Distribution of the number of cytosines per context for regions vs. 100 bp bins. The median for regions is 8, 9, and 31 for CG, CHG, and CHH. Median numbers per bin are 4, 5, and 26. **B** Distribution of the density of cytosines per context in regions vs. 100 bp bins. The median for regions is 0.11 for CG, 0.10 for CHG, and 0.30 for CHH, while for bins it is 0.04, 0.05, and 0.26. The modality of C density in regions is caused by Singlets and Duplets, which consist of 1–3 Cs in 1–3 bps. **C** Percentages of annotations overlapping with regions per context. The dots represent the percentages of single cytosines. **D** Mean methylation levels and **E** standard deviation per region for all MA lines, colored by whether they were called methylated, unmethylated, or intermediate.

After partitioning the genome based on C density and distance following the strategy outlined above, we found 430554 CG regions, 473588 CHG regions, and 804835 CHH regions in the *A. thaliana* genome (Genomic Coordinates in Table S4, S5, and S6). The median number of cytosines per region was 8 (max 66) for CG, 9 (max 66) for CHG, and 31 (max 97) for CHH (Fig. 3A). Accordingly, the median cytosine density per region (i.e., the ratio of base pairs which are C) for CG, CHG, and CHH were 0.11, 0.10, and 0.30, respectively (Fig. 3B). These observations indicate that most regions were made up of close cytosines.

For comparison, we partitioned the genome into 100 bp non-overlapping bins. On average, 100 bp bins contained fewer cytosines per bin and consequently lower cytosine densities than the regions (Fig. 3A, B). The median number of 4 CGs and 5 CHGs per bin is approximately half as high as for regions, while the median of 26 CHHs per bin is only slightly lower than in regions. The same is true for the median C densities, at 0.04, 0.05, and 0.26 for CG, CHG, and CHH. Moreover, 28% of CG and 21% of CHG bins were made up of only one or two cytosines on either strand, while only 10% and 7% of CG and CHG regions consisted of a pair of Cs (by construction, regions have at least 2 Cs). These higher percentages are relevant, as our aim is to group together close neighboring cytosines instead of constructing a large number of regions with only one or two Cs each. For CHH both the percentage of regions or bins with ≤2 CHHs were low, due to the relative abundance of CHHs throughout the genome (0.008% in bins and 3% in regions).

To study if the genome-wide epimutation rate estimation for regions was not skewed by an over-represented annotation, we calculated the proportion of regions that overlap with a given genomic annotation (Fig. 3C). These proportions closely resembled the proportions calculated for single cytosines (dots in Fig. 3C), which allows for meaningful comparisons of the epimutation rate estimates between regions and single Cs.

The constructed regions were applied to the WGBS data by summarizing the reads per region. Depending on the MA line, between 34.5% and 75.5% of regions fulfilled the filtering criteria of postMax ≥0.99 and a mean coverage of at least 3 reads per C in region. Lines MA1_1 and MA1_2 only retained 34.5–53.1% of their regions, while for MA1_3 and MA2_3 65.0–75.5% of regions met the filtering criteria (Table S1).

Finally, we explored the methylation levels and status calls per region. As expected, methylation levels (methylated reads/total reads) of most U regions were 0, while the levels of M regions peaked around 0.9 and methylation levels of I regions varied between 0.1 and 0.7 (Fig. 3D). Next, we studied the extent of the intra-region heterogeneity by calculating a standard deviation over cytosine-level methylation status calls (U, I, and M, represented as 0, 0.5, and 1) for each region. The I regions showed larger standard deviations compared to U and M regions (Figs. 3E and S4B, D), suggesting that U and M regions were very homogeneous, while I regions were a mixture of methylated and unmethylated cytosines. However, the number of I regions was very low in the genome, as on average only ≈8%, 4% and 9% of CG, CHG, and CHH regions were I. U regions made up the largest proportion of regions in all contexts (≈67%, 91%, and 89% for CG, CHG, and CHH, respectively), while the percentage of methylated regions were highest in CG (≈25%), but low in CHG (≈6%) and CHH (≈2%) (Figs. 3D and S4A, C). These results revealed that the constructed regions cluster sets of homogeneous cytosines that reflect the known genome-wide methylation levels as well as the same proportions of genomic annotations as single cytosines.

### Genome-wide methylation divergence and epimutation rates

**Fig. 4:**
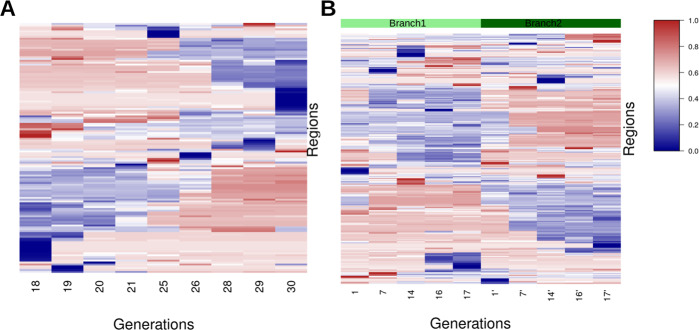
Heatmap of region-wise mean methylation levels over multiple generations. **A** All regions from MA line MA1_3 that were assigned both U and M states in at least one individual plant. **B** Regions from MA line MA2_3. The regions were clustered using hierarchical clustering.

We explored whether the regions changed methylation status over time in the same way as single Cs (van der Graaf et al. 2015). We visualized methylation status per region in the more densely sampled pedigrees, MA1_3 and MA2_3 (Fig. 1). A number of 135 and 277 regions changed methylation status over time and 60% and 48% of these regions remained stable in subsequent generations after the methylation change had taken place, respectively (Fig. 4).

**Fig. 5:**
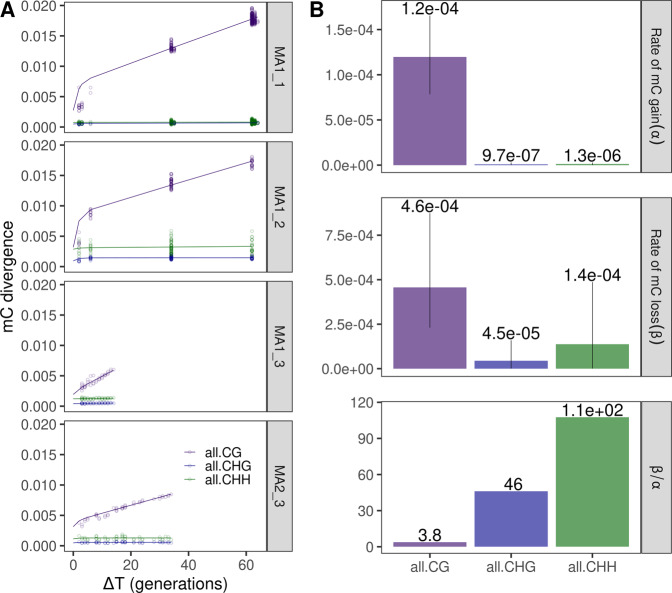
Genome-wide divergence and epimutation rates for CG, CHG, and CHH regions. **A** mC divergence over generation time for the four MA line pedigrees. **B** Mean epimutation rates per context. Error bars represent the maximal and minimal estimates out of the four MA line pedigrees.

To quantify these methylation changes genome-wide, we calculated methylation divergence over time using Eq. (1) and estimated epimutation rates using the model outlined above. We observed that, in all pedigrees, methylation divergence per region in CG context accumulated over time, i.e., two plants which had been selfed separately for a longer time (with larger Δ*t*) displayed a higher methylation divergence between them than two plants that were closer to each other in divergence time (Fig. 5A). The average CG gain rate α was estimated at 1.2 · 10^−4^ (range: 7.8 · 10^−5^–1.7 · 10^−4^), while the average CG loss rate *β* was estimated at 4.6 · 10^−4^ (range: 2.3 · 10^−4^–8.7 · 10^−4^) (Fig. 5B).

Although the divergence profiles of CHG and CHH regions seemed to represent very little accumulation of methylation changes over time, both neutral accumulation model fits were found to be significantly different from the null model of no accumulation (Fig. 5A). In accordance with the much lower divergence of CHG and CHH, the epimutation rates were smaller than for CG (for CHG α = 9.6 · 10^−7^ (range: 5.0 · 10^−8^–2.9 · 10^−6^), β = 4.4 · 10^−5^ (range: 6.0 · 10^−7^–1.6 · 10^−4^); for CHH α = 1.3 · 10^−6^ (range: 4.2 · 10^−9^–2.0 · 10^−6^), *β* = 1.4 · 10^−4^ (range: 7.4 · 10^−7^– 4.9 · 10^−4^), Fig. 5B). The higher *β*/*α* ratios in CHG (46) and CHH (112) in comparison to CG (3.8) reflect the lower genome-wide proportion of methylated regions (see above, Fig. S4A, C). The low observed epimutation rates translated into only 33 and 87 regions undergoing a transition from (on average in the four MA lines, for CHG and CHH respectively), compared to an average of 687.5 CG regions which changed methylation state in the same populations. This low accumulation led to epimutation rates which were very variable across MA lines. We, therefore, abstained in the following from splitting these low numbers of regions even further to study annotation-wise epimutation rates in the CHG and CHH contexts.

We tested if a methylation divergence model that considers selection for U or M methylation states fits the data better than the neutral model of epiallele inheritance. Model comparisons revealed that ABneutral provided the best fit to the CG as well as to the CHG and CHH data, indicating that the gain and loss dynamics were neutral in all contexts, at least globally (Table S3). The only exceptions were contexts CHG and CHH in MA line MA2_3 where models ABuu and ABmm were slightly favored (Table S3); however, this may be an artifact of few and noisy data points.

Lastly, we estimated context-specific epimutation rates using the 100 bp bin-based approach (Fig. S6). These rates were comparable to the region-based approach, with methylation gain rates being slightly lower and loss rates higher than for regions. By estimating epimutation rates for regions with a higher density and higher number of cytosines per region compared to bins, we minimized the possibility of overemphasizing the impact of single cytosines on the epimutation rate estimates (as 28% of bins are made of one or two cytosines). In fact, epimutated bins contained on average considerably fewer cytosines than epimutation regions (Fig. S6C). The similarity between region- and 100bp-bin based approaches suggests that the pace of methylation change accumulation is to a large extent robust to variations of the region definition, probably due to a large amount of bins and regions in the genome.

### Estimation of feature-specific epimutation rates

In addition to genome-wide CG methylation divergence, we also investigated different factors that might have an effect on the rate of methylation change accumulation, namely (1) characteristics of the constructed regions, (2) different genomic annotations, and (3) chromosomal organization.

**Fig. 6:**
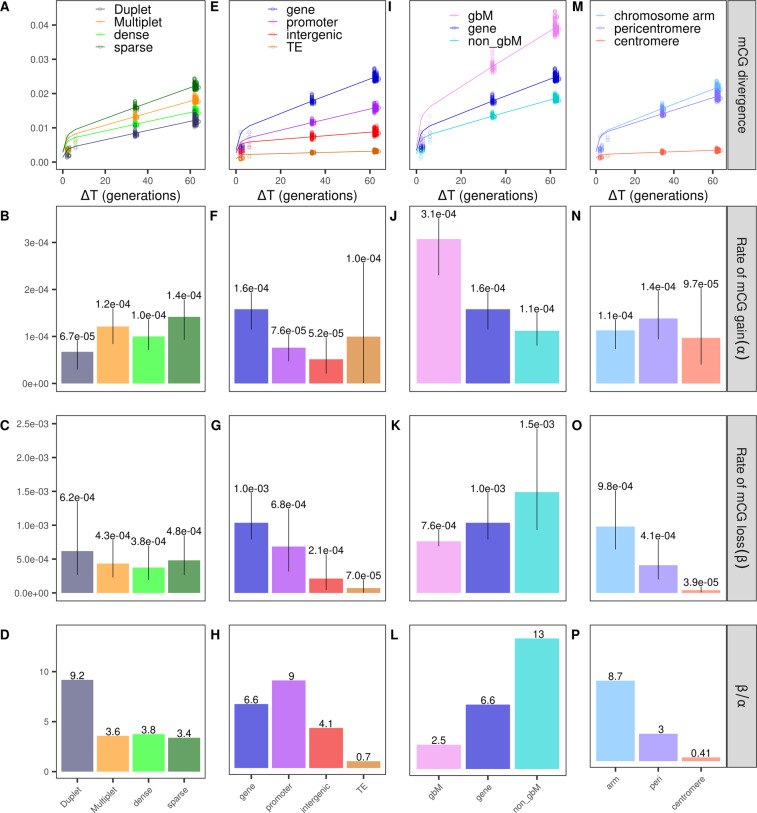
mCG divergence profiles of MA1_1 and mean epimutation rate estimates for different genomic features. Variations of α and β estimates between MA lines are represented as error bars. All rates per MA line and genomic features are found in Table S1. Divergence profiles for all MA lines can be seen in Fig. S7. **A**–**D** “Duplet” regions (slategrey) vs. regions with “Multiple” CGs (orange). The regions with multiple CGs were further separated into regions with high (“dense”, bright green) and low density (“sparse”, dark green). **E**–**H** Genes (blue), promoters (purple), intergenic regions (red), and TEs (darkorange). **I**–**L** Gene-body methylated genes (violet) vs. non-gbM genes(turquoise). **M**–**P** Chromosome arms (light blue), pericentromeres (lilac), centromeres (salmon).

To study whether the characteristics of the constructed regions had an effect on methylation loss and gain dynamics, we estimated the epimutation rates for regions of differing size and density. The regions were divided into duplets (2 CGs on opposite strands) and multiplets (regions with multiple CGs), which were further subdivided into dense and sparse regions based on their C density, as described above. The methylation divergence (Fig. 6A) showed a similar accumulation slope for regions of different characteristics, despite the fact that the different regions had variable intercepts (which is attributable to measurement noise (van der Graaf et al. 2015)). Consequently, the epimutation rates were also very similar across the different region types, indicating that region size and cytosine density had no major effect on epimutation rates. The same held true when estimating epimutation rates for multiplets that were shorter and longer than the median region size (Fig. S8). However, we did notice a tendency for duplets to lose more and gain less methylation compared to multiplets, resulting in a higher *β*/*α* ratio. This preferential methylation in CG-rich regions has also been observed using WGBS (Cokus et al. 2008).

Furthermore, we calculated epimutation rates for regions overlapping with genes, promoters, intergenic regions, and TEs separately. As previously observed for single CGs (van der Graaf et al. 2015), the annotation for which regions accumulated the most transgenerational changes was genes, followed by promoters. For intergenic regions and TEs, on the other hand, the divergence profiles showed very little accumulation (Fig. 6E). The average estimated CG epimutation rates are displayed in Fig. 6F–H (rates per MA line in Table S1).

Genes exhibited the highest epimutation rates and a ratio *β*/*α* of 6.6, which is consistent with the fact that most genes are unmethylated. This could in part, be attributed to the inability of genic methylation to be recovered once severely compromised (Stroud et al. 2013). Moreover, we split genes into gene body methylated (gbM) genes and non-gbM genes using the classification from Bewick et al. (2016) (gbM: mCG enrichment in the transcribed region and depletion of mCG at transcription start and stop site). As expected, this resulted in regions overlapping non-gbM genes being unmethylated at a higher percentage (87%, averaged over the considered MA lines) than those overlapping gbM genes (54%). Interestingly, this more detailed analysis revealed that the accumulation of methylation divergence was more rapid in regions located in gbM genes than in regions overlapping non-gbM genes (Fig. 6I). On the other hand, the epimutation rates of gbM and non-gbM genes resembled a trade-off between methylation gain and loss: while gbM genes gained methylation almost three times as fast as non-gbM genes, non-gbM genes lost methylation at about twice the rate as gbM genes (Fig. 6J, K). The ratio *β*/*α* of non-gbM genes, which was higher than in any other genomic feature (Fig. 6L), implies that while non-gbM genes were largely kept free of methylation, gbM genes remained considerably more methylated than promoters and intergenic regions, reflecting the higher methylation levels of gbM genes in comparison to non-gbM genes.

Apart from the non-gbM genes, the highest *β*/*α* ratio was estimated for promoters, where methylation loss was ≈9 times higher than methylation gain. This reflects that only 5% of expressed genes were found to be methylated within their promoters (Zhang et al. 2006). TEs, on the other hand, was the only annotation for which gaining methylation was more probable than losing it (*β*/*α* = 0.7, Fig. 6H). High levels of CG methylation in TEs have already been reported as highly conserved and differential methylation of CGs was found to be depleted in TEs and enriched in genes (Becker et al. 2011, Schmitz et al. 2013b). The same underrepresentation of CG-DMPs in TEs was observed in soybeans (Schmitz et al. 2013a). This faithfully maintained DNA methylation is a product of targeting by the de novo methylation machinery, stemming from the high-risk TEs pose to genome integrity (Law and Jacobsen 2010; Stroud et al. 2013).

Epimutation rates were also calculated for 5′- and 3′-UTRs, although the small number of regions overlapping these annotations made the estimates very variable across MA lines, and especially the MA lines with a smaller number of samples (MA1_3 and MA2_3) showed comparatively large standard errors (Table S1). We did observe that the epimutation rates of 3′-UTRs were higher than the rates of 5′-UTRs in all MA lines. Within gene bodies, the 3′ end is the most enriched in CG-DMRs (Schmitz et al. 2011), which could explain the higher epimutation rates in these regions. Both 5′- and 3′-UTRs displayed very high *β*/*α* ratios (44-87 for 5′- and 10-28 for 3′-UTRs). This tendency towards methylation loss hints at a mechanism that keeps these highly conserved sequences of 5′- and 3′-UTRs, which are critical for controlling mRNA translation and regulating rRNA turnover, respectively, in an unmethylated state (Mignone et al. 2002).

Finally, we explored epimutation rates per chromosome region. mCG divergence was highest for regions in chromosome arms, closely followed by pericentromeres, while centromeres displayed almost no transgenerational changes (Fig. 6M). Similar observations were also made by Schmitz et al. (2011), who reported depletion of CG-DMRs in pericentromeres and centromeres, and by van der Graaf et al. (2015) who for single cytosines found higher mCG divergence in chromosome arms. Estimation of gain and loss epimutation rates showed that all chromosome regions had similar mCG gain rates (Fig. 6N), but the mCG loss was highest in chromosome arms while lowest in centromeres (Fig. 6O). Therefore, while chromosome arms appeared to lose methylation a lot quicker than they gain it, centromeres were the genomic feature that revealed the strongest preference for methylation gain over loss out of all the investigated genomic features (*β*/*α* = 0.41, Fig. 6P). The prevalence of high mCG conservation in centromeres coincides with the distribution of both TEs/repeats and heterochromatin along the genome (Cokus et al. 2008; Schmitz et al. 2013b; van der Graaf et al. 2015), suggesting either confounding with annotation or the influence of heterochromatin-specific methylation factors such as DDM1 (Stroud et al. 2013).

**Fig. 7:**
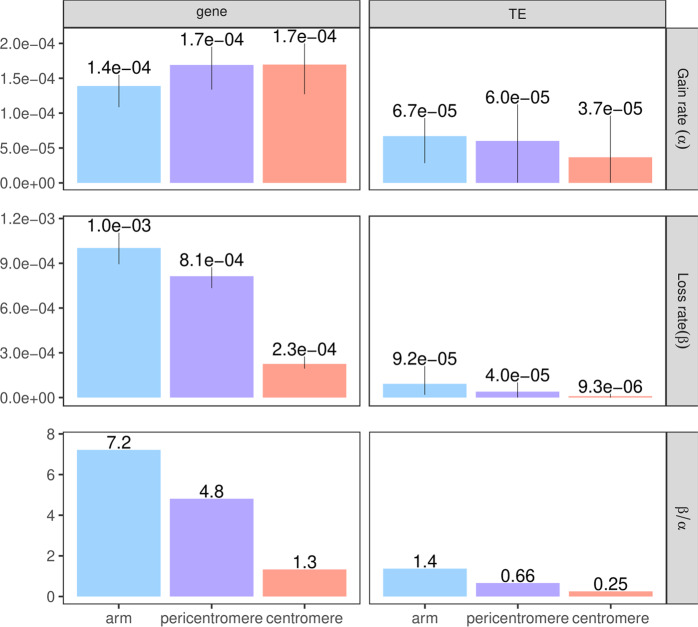
Epimutation rates for TEs and genes in three different chromosome regions. Chromosome arms (indigo), pericentromeres (violet), centromeres (salmon). MA line MA1_3 is not shown here as it has a very small number of samples and epimutation rates for this category, with a small number of regions to test, which leads to noisy estimates.

To explore the extent of the confounding between annotations and chromosome regions, epimutation rates were estimated for TEs and genes in chromosome arms, pericentromeres, and centromeres separately. From the results, it stands out that, in general, a genomic annotation accumulated more epigenetic changes when it was located in chromosome arms compared to centromeres. TEs in chromosome arms even showed a slightly higher mCG loss than gain. In addition, regardless of chromosome regions, genes were always associated with higher epimutation rates than TEs (Fig. 7). Therefore differences in epimutation rates specific to chromosome regions cannot be simply caused by the distribution of TEs vs. genes along the genome. More probably chromatin architecture also plays a relevant role in shaping epimutation rates and epigenomic divergence over time.

As for the genome-wide estimations, we compared the methylation divergence fit for the models with and without selection. In all lines and all annotations, the model without selection could fit the data best (f1-scores and *p*-values in Table S2). Therefore, if present, genome-wide selection against a methylation state is very low.

## Discussion

In this paper, we provide a comprehensive study of region-wise epimutation rates in the model plant *A. thaliana*. After the construction of methylation regions, we found that epimutation rates for regions were in the same order of magnitude as those observed for single CGs (van der Graaf et al. 2015). This finding indicates that over generation time coordinated changes of methylation involving neighboring cytosines occur at a comparable rate as individual cytosine changes. These estimations can have far-reaching implications since changes in the methylation status of clusters of cytosines may have more relevant effects on phenotypes than changes in single isolated cytosines, especially when considering regulatory elements such as promoters. Moreover, our model comparison between no selection and selection models showed that, overall, the observed methylation accumulation profiles could be best explained by a model that assumes no genome-wide selection pressure against losing or gaining methylation. Evidence of no selection has been observed in natural populations of *A. thaliana* using methylation side frequency spectrum techniques (Vidalis et al. 2016), but only for single cytosines.

We have shown that the rates of region-level mCG changes, while only marginally dependent on region size and cytosine density, strongly depended on genome annotation and are consistently highest in genes and lowest in TEs. This hierarchy of epimutation rates per annotation is not only preserved from single CG estimates in *A. thaliana* (van der Graaf et al. 2015) but is also conserved in mitotic studies of epimutation rates in *Populus trichocarpa* (Hofmeister et al. 2020). This suggests that the hierarchy of epimutations is not a product of methylation reinforcement events during seed development but is conserved by DNA methylation maintenance pathways during mitotic cell division upon somatic development in plants (Hofmeister et al. 2020; Johannes and Schmitz 2019).

The function of CG methylation in genes, especially gene body methylation still remains elusive, despite hypotheses linking it to transcription (Bewick and Schmitz 2017). Consistent with the accumulation of gbM over time observed by Bewick and Schmitz (2017), the estimated *α* rates showed that regions in gbM genes gained methylation much more rapidly than regions in non-gbM genes (and any other annotation) whereas regions in non-gbM genes lost methylation quicker (*β*) (Fig. 6J). Takuno and Gaut (2013) reported that although the classification of gbM genes was conserved among different *A. thaliana* MA lines as well as between *O. sativa*, *Z. Mays*, and *B. distachyon*, the methylation of individual CG sites were highly polymorphic, which is consistent with our observed high epimutation rates. The authors proposed a model by which the conservation of gbM across species implies functional relevance and that individual CG sites of methylation may vary without compromising proper gene function as long as the methylation level per gene is kept above a certain threshold. On the other hand, the higher rate of methylation accumulation has been hypothesized to be a passive byproduct of the errant properties inherent to the heterochromatin machinery, rather than a symptom of functional relevance (Bewick and Schmitz 2017; Wendte et al. 2019).

Estimation of epimutation rates at chromosome arms, centromeres, and pericentromeres separately showed that epimutation rates were heavily dependent on chromosomal location, with rates that were lowest at centromeres and highest in arms. Although chromosome location is partially confounded with annotation (as arms are gene-rich and centromeres are TE-rich), this could not completely explain the observed trend, as epimutation rates for TEs in chromosome arms were higher than epimutation rates for genes in centromeres (Fig. 7). This means that—independently of annotation-specific targeting takes place in centromeres in comparison to chromosome arms. Indeed, TEs in chromosome arms near active genes were shown to be silenced through RdDM (RNA directed DNA METHYLATION), while the silencing of TEs in gene-poor centromeres is dependent on DDM1 (Zemach et al. 2013). Since RdDM requires the nucleosome remodeler DRD1 to maintain DNA methylation and DRD1 remodels heterochromatic nucleosomes less effectively (Zemach et al. 2013), this may lead to less faithfully maintained methylation in TEs located in chromosome arms compared to TEs in centromeres.

Also using *A. thaliana* Col-0 MA lines from Shaw et al. (2000), Weng et al. (2019) recently published a genome-wide mutation rate of 6.95 · 10^−9^ per site and generation. The genome-wide epimutation rates for CG regions were five orders of magnitude higher than this mutation rate. This finding indicates that similarly to single Cs, region-level epimutations occur independently of genetic mutations and provide a separate source of genome evolution and diversity (Monroe et al. 2020). Our results have major implications for understanding how LD between genetic variants and DMRs evolves over time, and substantiate the need for methylation-based EWAS in plants.

**Fig. 8:**
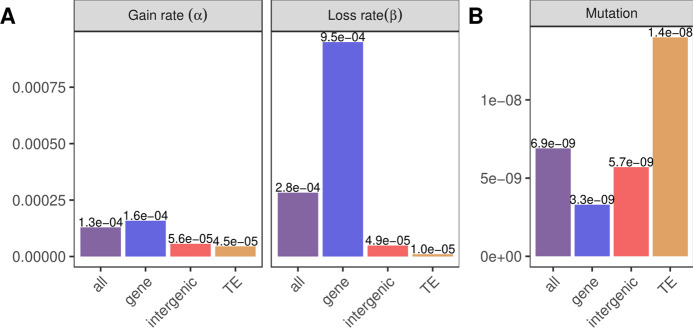
Comparison of region-level epimutation rates with genetic mutation rates. **A** Gain and loss rates calculated for MA line MA1_1. Epimutation rates of all CG regions (purple) and subdivided into regions overlapping genes (blue), intergenic regions (red) and TEs (orange). **B** Genetic mutation rates calculated by Weng et al. (2019) genome-wide (“all"), as well as for genes, intergenic regions and TEs.

Weng et al. (2019) also calculated, for the first time, mutation rates for different genomic annotations, distinguishing between TEs, genic and intergenic regions. The mutation rate for TEs (1.36 · 10^−8^) was nearly four times higher than that of genes (3.35 · 10^−9^) and the mutation rate of intergenic regions was almost twice as high as the genic mutation rate (5.75 · 10^−9^). These mutation values displayed the complete opposite trend as observed for epimutations, where changes were lowest in TEs and highest in genes (Fig. 8). The same trend was observed when splitting the genome into chromosome arms and (peri-)centromeres: The mutation rates per chromosome region reported by Weng et al. (2019) showed that the mutation rate was more than twice as high in (peri-)centromeric regions as in chromosome arms. These observations reflect large genome-wide differences between genetic and epigenetic divergence and have implications for understanding genome-methylome co-evolution.

This study presents the first estimates for region-level epimutation rates for *A. thaliana*. The observed methylation changes and subsequent epimutation rates showed that methylation changes of clusters of cytosines accumulate almost as fast as in single cytosines, suggesting that they are independent of genetic change. In addition, we have shown that annotations (genes, promoters, TEs) and chromosome regions are highly predictive of the epimutation rates. Genes and chromosome arms feature the highest accumulation of region methylation changes, while TEs in centromeres have the lowest epimutation rates. Comparing our epimutation rate estimates to the mutation rates estimated by Weng et al. (2019) we observed an anti-correlation between mutation and epimutation rates.

## Data archiving

The data, in the form of processed count matrices, was downloaded from GEO with the accession number GSE64463, where the lines MA1_3 and MA2_3 are located in the main GSE matrix and the lines MA1_1 and MA1_2 can be found in the supplementary files. MA1_3 and MA2_3 are described in detail by van der Graaf et al. (2015), while MA1_1 is described in Becker et al. (2011) and MA1_2 in Schmitz et al. (2011).

## Supplementary information

Supplementary Material

Table S3

Table S4

Table S5

Table S6

## References

[CR1] Becker C, Hagmann J, Müller J, Koenig D, Stegle O, Borgwardt K (2011). Spontaneous epigenetic variation in the *Arabidopsis thaliana* methylome. Nature.

[CR2] Bewick AJ, Ji L, Niederhuth CE, Willing EM, Hofmeister BT, Shi X (2016). On the origin and evolutionary consequences of gene body DNA methylation. Proc Natl Acad Sci USA.

[CR3] Bewick AJ, Schmitz RJ (2017). Gene body DNA methylation in plants. Curr Opin Plant Biol.

[CR4] Cokus SJ, Feng S, Zhang X, Chen Z, Merriman B, Haudenschild CD (2008). Shotgun bisulphite sequencing of the *Arabidopsis* genome reveals DNA methylation patterning. Nature.

[CR5] Cubas P, Vincent C, Coen E (1999). An epigenetic mutation responsible for natural variation in floral symmetry. Nature.

[CR6] Gallusci P, Hodgman C, Teyssier E, Seymour GB (2016). DNA methylation and chromatin regulation during fleshy fruit development and ripening. Front Plant Sci.

[CR7] Ganguly DR, Crisp PA, Eichten SR, Pogson BJ (2017). The arabidopsis DNA methylome is stable under transgenerational drought stress. Plant Physiol.

[CR8] Hofmeister BT, Denkena J, Colomé-Tatché M, Shahryary Y, Hazarika R, Grimwood J (2020). A genome assembly and the somatic genetic and epigenetic mutation rate in a wild long-lived perennial *Populus trichocarpa*. Genome Biol.

[CR9] Hofmeister BT, Lee K, Rohr NA, Hall DW, Schmitz RJ (2017). Stable inheritance of DNA methylation allows creation of epigenotype maps and the study of epiallele inheritance patterns in the absence of genetic variation. Genome Biol.

[CR10] Jiang C, Mithani A, Belfield EJ, Mott R, Hurst LD, Harberd NP (2014). Environmentally responsive genome-wide accumulation of de novo *Arabidopsis thaliana* mutations and epimutations. Genome Res.

[CR11] Johannes F, Colot V, Jansen RC (2008). Epigenome dynamics: a quantitative genetics perspective. Nat Rev Genet.

[CR12] Johannes F, Schmitz RJ (2019). Spontaneous epimutations in plants. N Phytol.

[CR13] Kawakatsu T, Huang S-SC, Jupe F, Sasaki E, Schmitz RJ, Urich MA (2016). Epigenomic diversity in a global collection of *Arabidopsis thaliana* accessions. Cell.

[CR14] Law JA, Jacobsen SE (2010). Establishing, maintaining and modifying DNA methylation patterns in plants and animals. Nat Rev Genet.

[CR15] Mignone F, Gissi C, Liuni S, Pesole G (2002). Untranslated regions of mRNAs. Genome Biol.

[CR16] Monroe JG, Srikant T, Carbonell-Bejerano P, Exposito-Alonso M, Weng M-L, Rutter MT et al. (2020) Mutation bias shapes gene evolution in *Arabidopsis thaliana*. bioRxiv 2020.06.17.156752.

[CR17] Ong-Abdullah M, Ordway JM, Jiang N, Ooi SE, Kok SY, Sarpan N (2015). Loss of Karma transposon methylation underlies the mantled somaclonal variant of oil palm. Nature.

[CR18] Ossowski S, Schneeberger K, Lucas-Lledó JI, Warthmann N, Clark RM, Shaw RG (2010). The rate and molecular spectrum of spontaneous mutations in *Arabidopsis thaliana*. Science.

[CR19] Robinson JT, Thorvaldsdóttir H, Winckler W, Guttman M, Lander ES, Getz G (2011). Integrative genome viewer. Nat Biotechnol.

[CR20] Schmitz RJ, He Y, Valdés-López O, Khan SM, Joshi T, Urich MA (2013). Epigenome-wide inheritance of cytosine methylation variants in a recombinant inbred population. Genome Res.

[CR21] Schmitz RJ, Matthew DS, Lewsey MG, O’Malley RC, Urich MA, Libiger O (2011). Transgenerational epigenetic instability is a source of novel methylation variants. Science.

[CR22] Schmitz RJ, Schultz MD, Urich MA, Nery JR, Pelizzola M, Libiger O (2013). Patterns of population epigenomic diversity. Nature.

[CR23] Seymour DK, Becker C (2017). The causes and consequences of DNA methylome variation in plants. Curr Opin Plant Biol.

[CR24] Shahryary Y, Symeonidi A, Hazarika RR, Denkena J, Mubeen T, Hofmeister B (2020). AlphaBeta: computational inference of epimutation rates and spectra from high-throughput DNA methylation data in plants. Genome Biol.

[CR25] Shaw RG, Byers DL, Darmo E (2000). Spontaneous mutational effects on reproductive traits of. Genetics.

[CR26] Stroud H, Greenberg M, Feng S (2013). Comprehensive analysis of silencing mutants reveals complex regulation of the Arabidopsis methylome. Cell.

[CR27] Takuno S, Gaut BS (2013). Gene body methylation is conserved between plant orthologs and is of evolutionary consequence. Proc Natl Acad Sci USA.

[CR29] Taudt A, Roquis D, Vidalis A, Wardenaar R, Johannes F, Colome-Tatché M (2018). METHimpute: imputation-guided construction of complete methylomes from WGBS data. BMC Genom..

[CR30] The Arabidopsis Information Resource (2018) ftp://ftp.arabidopsis.org/home/tair/Genes/TAIR10_genome_release/TAIR10_gff3/. on www.arabidopsis.org. accessed 30-August-2018.

[CR31] van der Graaf A, Wardenaar R, Neumann DA, Taudt A, Shaw RG, Jansen RC (2015). Rate, spectrum, and evolutionary dynamics of spontaneous epimutations. Proc Natl Acad Sci USA.

[CR32] Vidalis A, Zivkovic D, Wardenaar R, Roquis D, Tellier A, Johannes F (2016). Methylome evolution in plants. Genome Biol.

[CR33] Wendte JM, Zhang Y, Ji L, Shi X, Hazarika RR, Shahryary Y (2019). Epimutations are associated with chromomethylase 3-induced de novo DNA methylation. eLife.

[CR34] Weng M-l, Becker C, Hildebrandt J, Neumann M, Rutter MT, Shaw RG (2019). Fine-grained analysis of spontaneous mutation spectrum and frequency in *Arabidopsis thaliana*. Genetics.

[CR35] Xu G, Lyu J, Li Q, Liu H, Wang D, Zhang M et al. (2020) Adaptive evolution of DNA methylation reshaped gene regulation in maize. *bioRxiv* 1–25.

[CR36] Zemach A, Kim MY, Hsieh PH, Coleman-Derr D, Eshed-Williams L, Thao K (2013). The arabidopsis nucleosome remodeler DDM1 allows DNA methyltransferases to access H1-containing heterochromatin. Cell.

[CR37] Zhang H, Yazaki J, Sundaresan A, Cokus S, Chan W-L, Chen H (2006). Genome-wide high-resolution mapping and functional analysis of DNA methylation in arabidopsis. Cell.

