# Supplementary material for: Region-level epimutation rates in Arabidopsis thaliana
Source: Heredity (Edinb). 2021 May 8;127(2):190–202. doi: 10.1038/s41437-021-00441-w (PMC8322157; doi:10.1038/s41437-021-00441-w)
Supplement: Supplementary file 1 — Supplementary Material [file 41437_2021_441_MOESM1_ESM.pdf]

Region-level Epimutation Rates in *Arabidopsis Thaliana*: Supplementary Information

Johanna Denkena[1], Frank Johannes[2], Maria Colomé-Tatché[1*]

Author Details:

[1] Institute of Computational Biology, Helmholtz Zentrum München Neuherberg 85764, Germany

[2] Department of Plant Sciences, Hans Eisenmann-Zentrum for Agricultural Sciences, Technical University Munich, Freising, Germany

*Corresponding author: maria.colome@helmholtz-muenchen.de

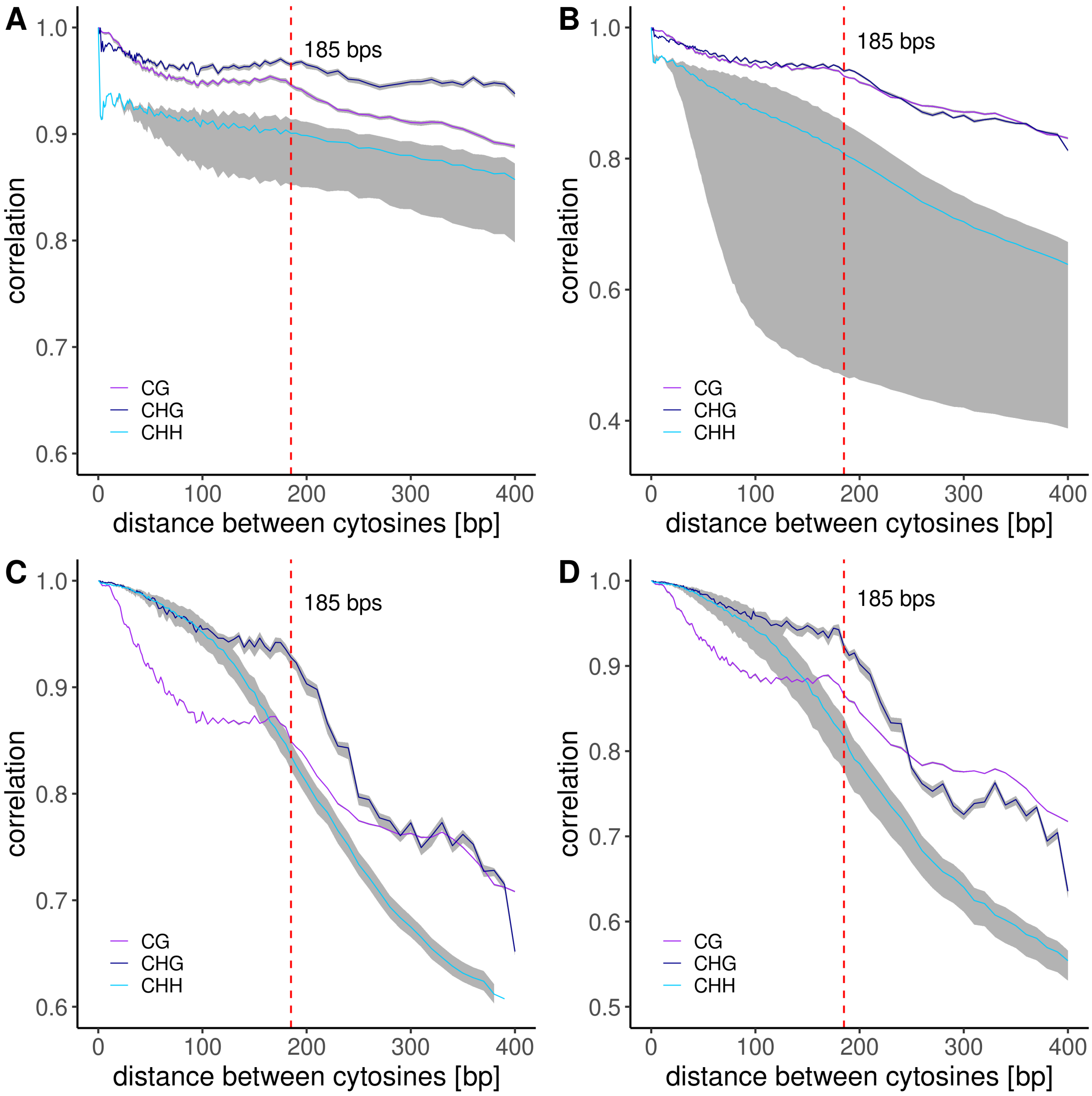

Figure S1: Autocorrelation of methylation states per context in MA line (**A**) MA1_1, (**B**) MA1_2, (**C**) MA1_3, (**D**) MA2_3. The grey shading around the lines represents the variation of the samples per MA line. The autocorrelation was calculated based on cytosines that fulfilled quality citeria (maximum posterior probability ≥ 0.9) in all samples per MA line. Over the first 200bp, the autocorrelation profiles differ between MA lines (especially CG). We assume this to be a batch effect between the data that was published in 2011 (MA1_1 and MA1_2) and 2015 (MA1_3 and MA2_3), which affects how coverage is distributed along the genome. To see how autocorrelation differs between MA lines when calculated using the same set of cytosines, see Figure S2.

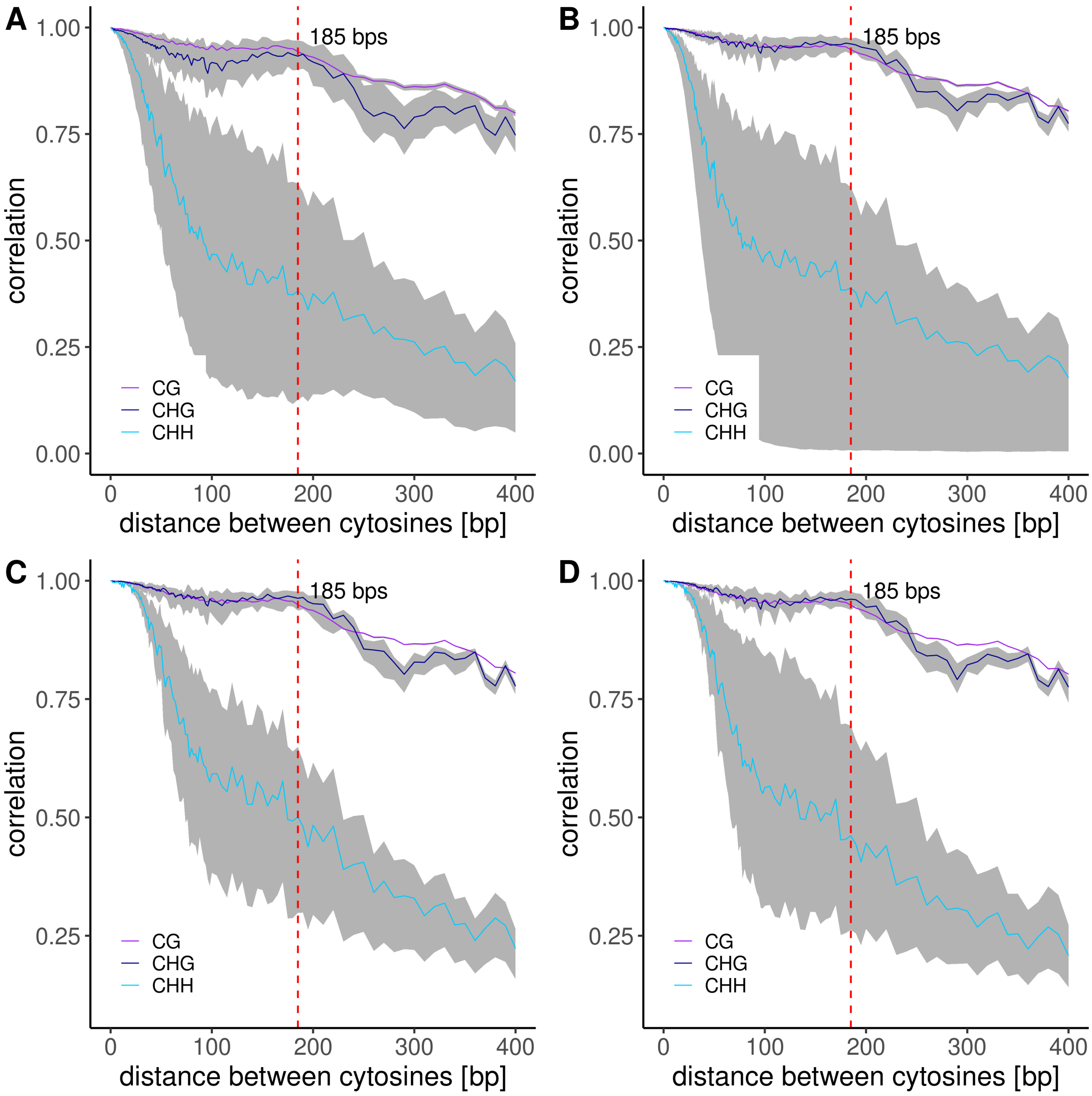

Figure S2: Autocorrelation of methylation states per context in MA line (**A**) MA1_1, (**B**) MA1_2, (**C**) MA1_3, (**D**) MA2_3. The autocorrelation was calculated based on cytosines that fulfilled quality citeria (maximum posterior probability ≥ 0.9) in all samples of all MA lines. The grey shading around the lines represents the variation of the samples per MA line.

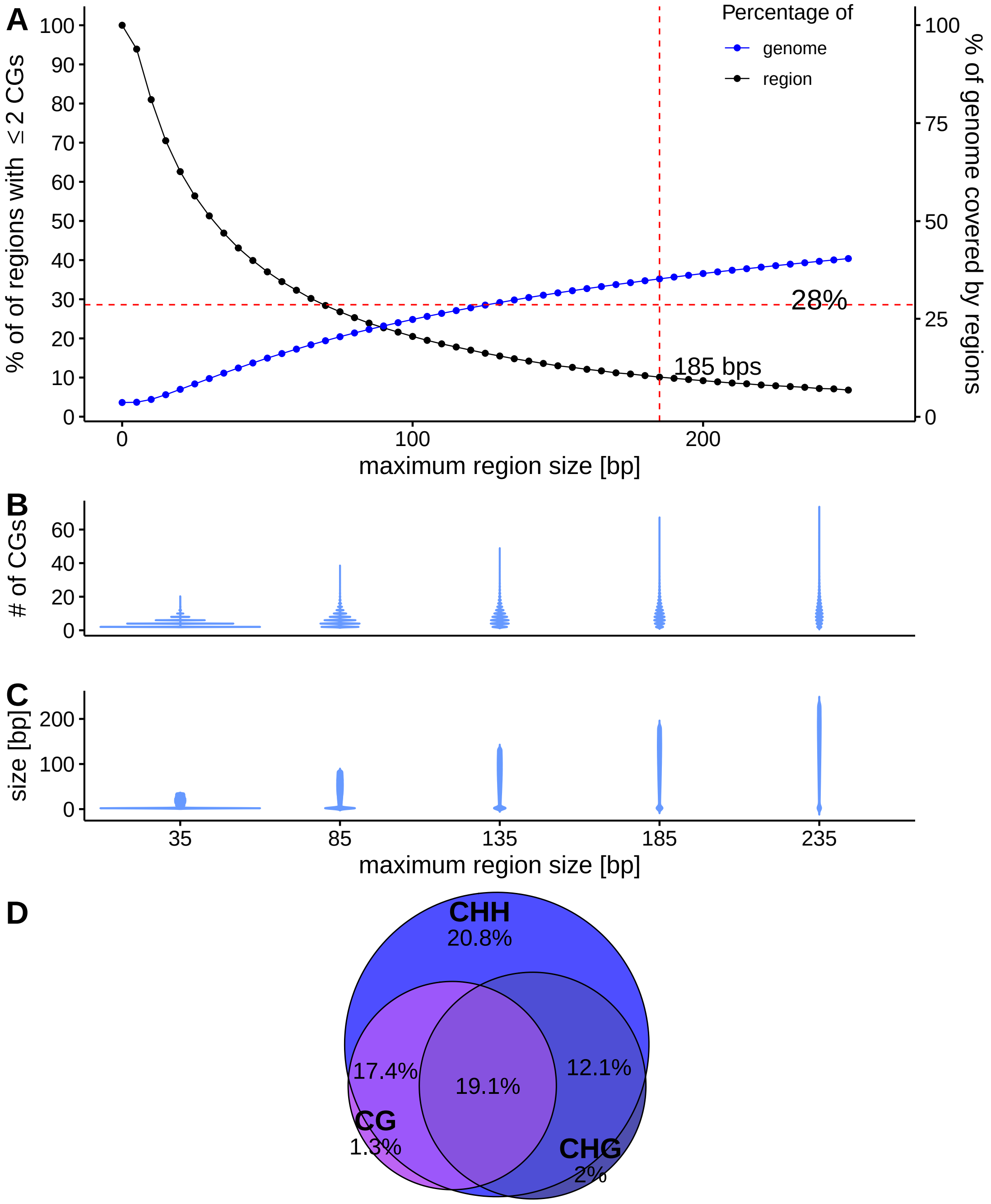

Figure S3: Impact of changing size cutoffs on region characteristics. (**A**) For different region size cutoffs the percentage of regions with only one or two Cs is plottet in black, while the percentage of the genomic space covered by these regions [bp] is represented in blue. For comparison, the red horizontal line represents the percentage of 100bp bins with 2 or 1 Cs. The red vertical line marks the chosen cutoff at 185bp, by which only 10% of regions contain two or less CG and 35.2% of the genome is covered. (**B**) Distribution of number of CGs per region, when constructed using different size cutoffs. (**C**) Distribution of region sizes[bp], when constructed using different size cutoffs. (**D**) Overlap of basepairs between CG, CHG and CHH regions (max. region size = 185bp) as percentage of bp in the genome.

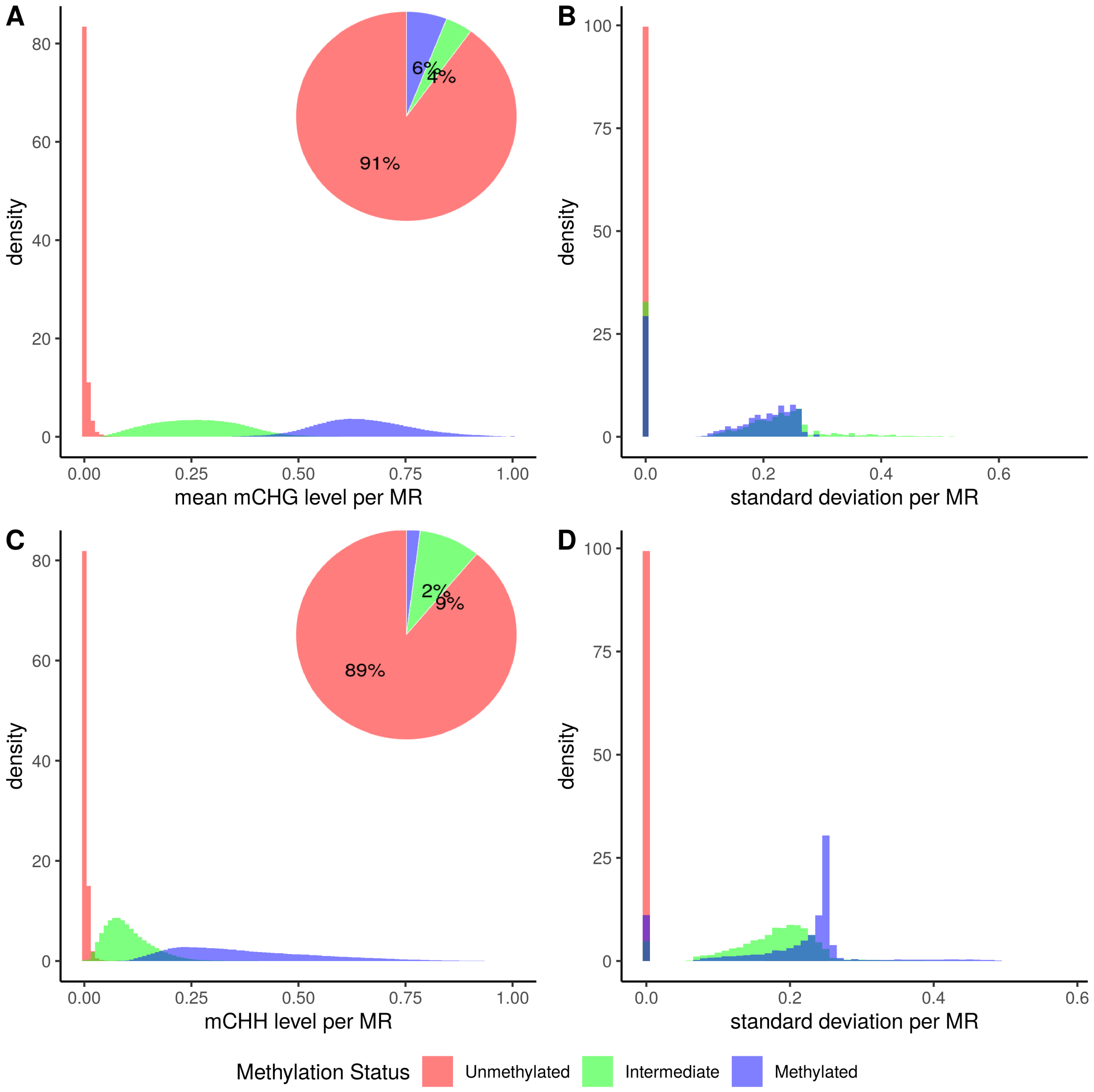

Figure S4: **(A)** Mean methylation levels per region for CHG context. **(B)** Standard deviation per region for CHG context. **(C)** Mean methylation levels per region for CHH context. **(D)** Standard deviation per region for CHH context. Colored by whether they were called as Methylated, Unmethylated or Intermediate.

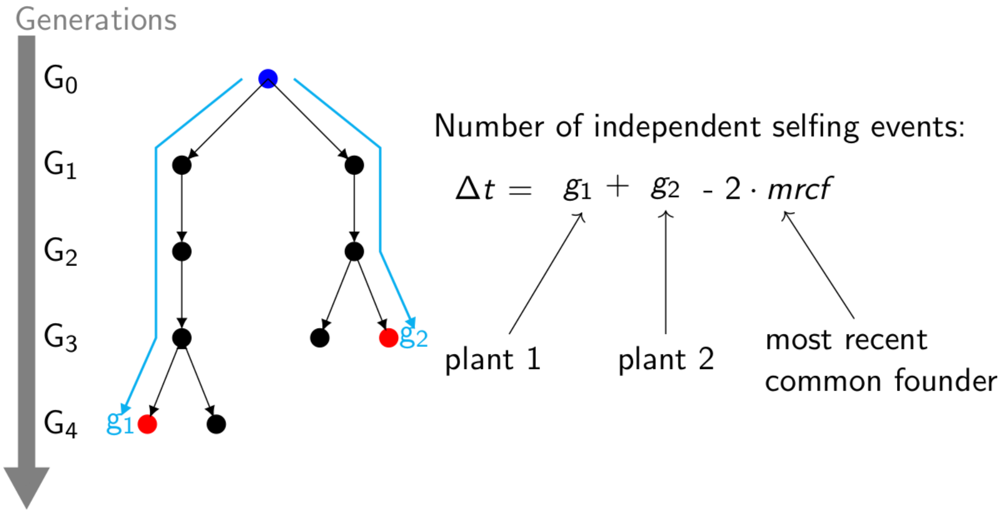

Figure S5: Schematic overview on how to calculate Δt.

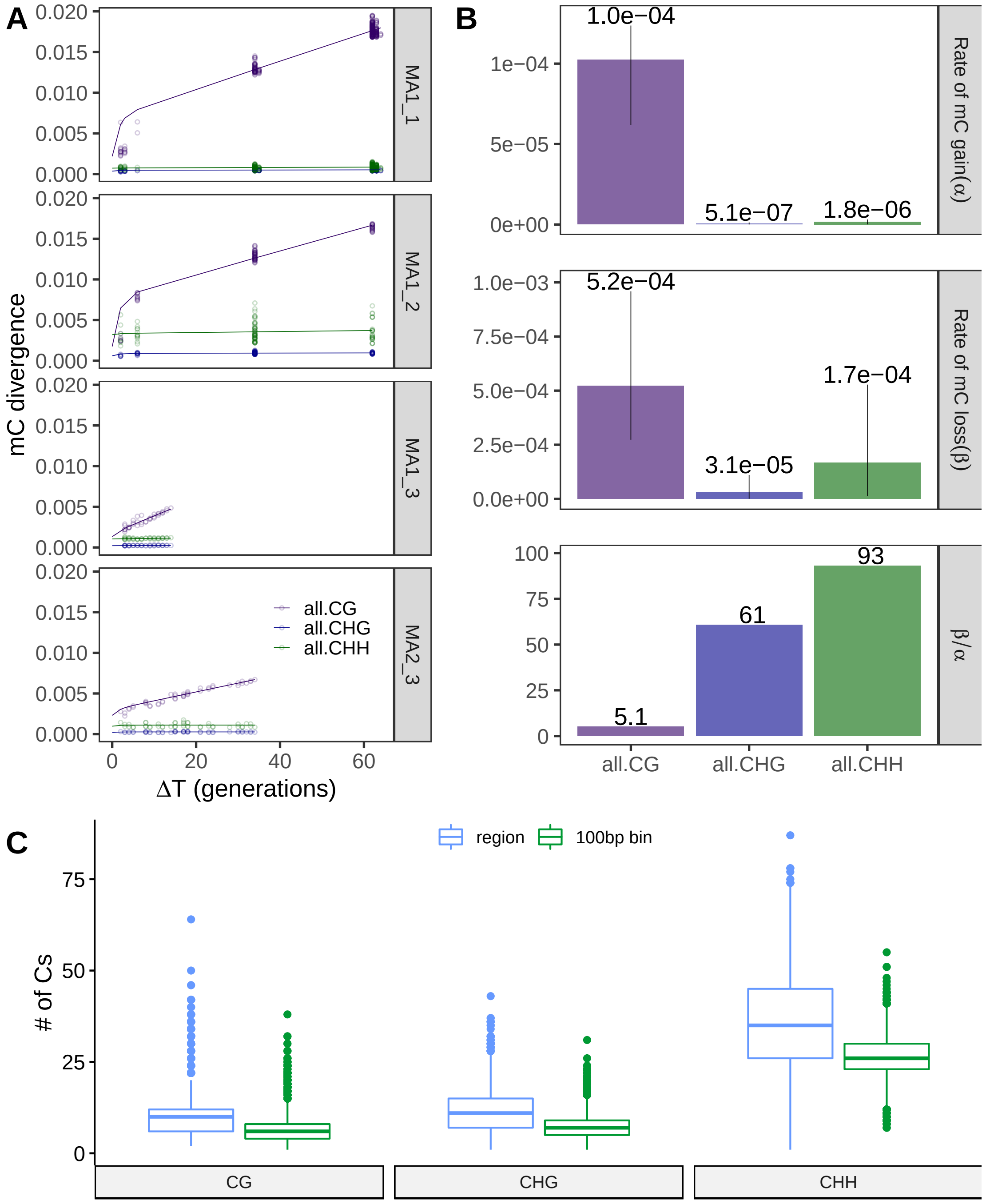

Figure S6: Divergence (**A**) and Epimutation Rates (**B**) for CG, CHG and CHH, calculated using the 100bp bin approach. **(C)** Number of cytosines per region/bin at which epimutations occur over all MA lines. Epimutated regions/bins are defined as having transitioned to a different methylation state at least once ($d_{ij,n} \neq 0$). The median number of cytosines for regions at which epimutations were observed is 10 (mean = 9.99), while that number is 6 for epimutated bins (mean = 5.99).

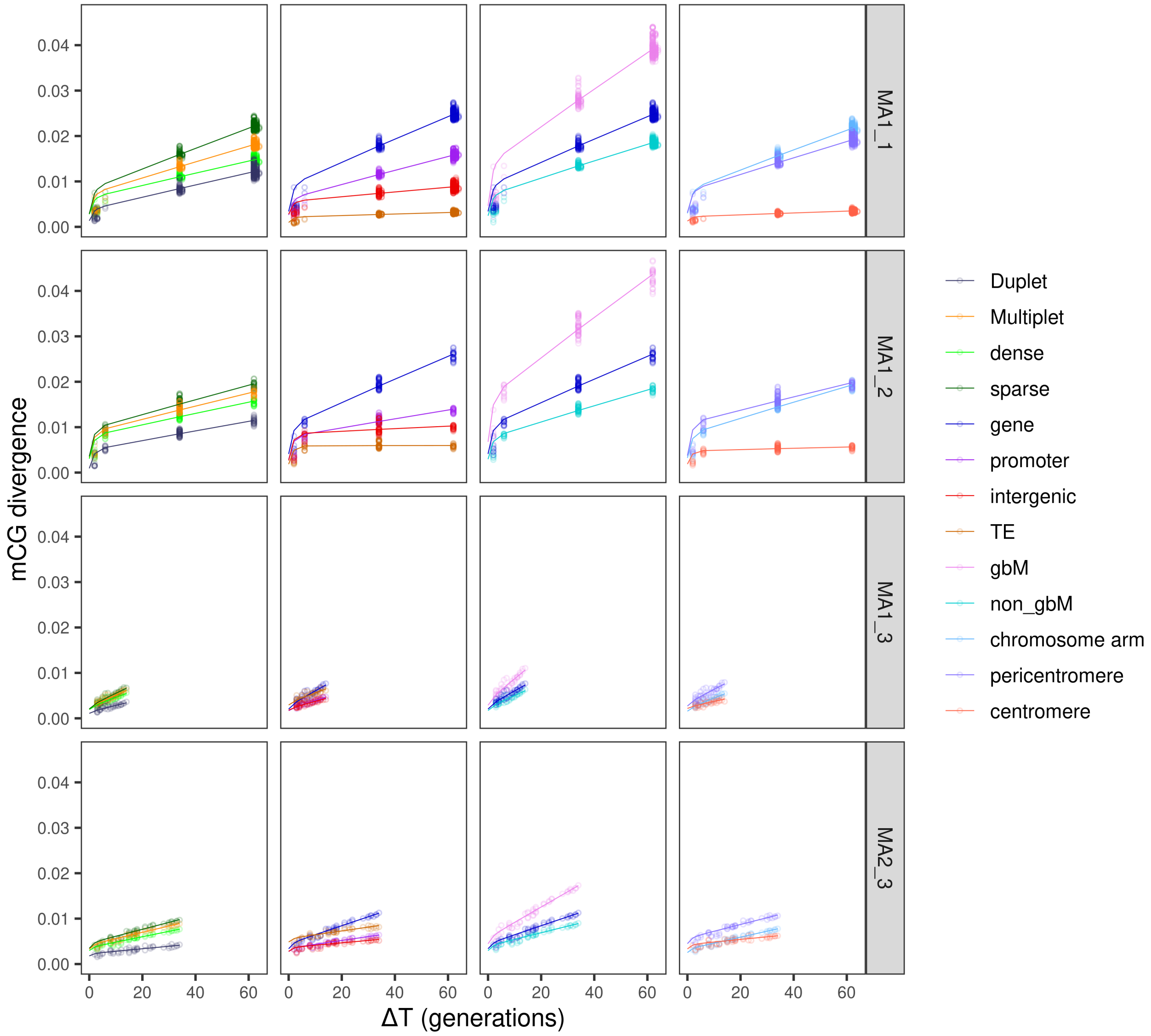

Figure S7: Divergence for all investigated genomic features and MA line pedigrees.

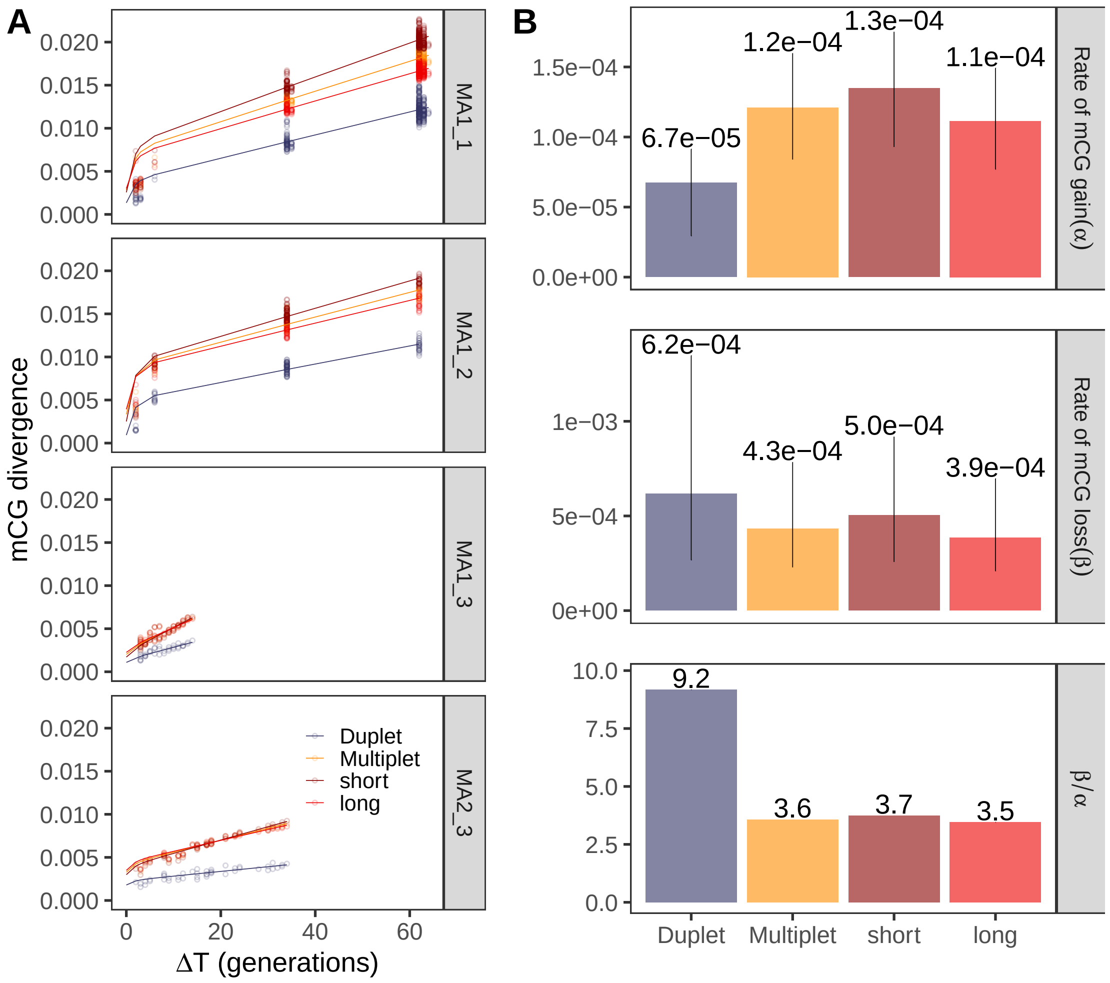

Figure S8: Divergence (**A**) and Epimutation Rates (**B**) for Duplets, Multiplets and Multiplets separated into regions shorter (”short”) and longer (”long”) than the median region size.

Context	MA	filtered[freq]	filtered[%]	meanCoverage
CG	MA1_1	197737	45.93	9.87
CG	MA1_2	228934	53.17	7.38
CG	MA1_3	325112	75.51	5.64
CG	MA2_3	298546	69.34	5.31
CHG	MA1_1	209291	44.19	9.51
CHG	MA1_2	233708	49.35	6.95
CHG	MA1_3	355326	75.03	5.62
CHG	MA2_3	330282	69.74	5.24
CHH	MA1_1	277806	34.52	7.63
CHH	MA1_2	311989	38.76	6.36
CHH	MA1_3	571015	70.95	5.63
CHH	MA2_3	523302	65.02	5.29

Table S1: Quality Control for CG, CHG and CHH regions per MA line. Frequency and Percentages of regions fulfilling both the posteriorMax >= 0.99 and the minimal mean coverage per region criterion relative to the total number of regions constructed from the reference genome. The column "meanCoverage" relays the mean number of reads per regions divided by the number of cytosines per regions for the regions specified in the left column.

	type	MA1_1.rates	MA1_2.rates	MA1_3.rates	MA2_3.rates	MA1_1.SE	MA1_2.SE	MA1_3.SE	MA2_3.SE
genome-wide		$1.29\cdot10^{-4}$	$1.06\cdot10^{-4}$	$1.66\cdot10^{-4}$	$7.84\cdot10^{-5}$	$2.35\cdot10^{-6}$	$4.10\cdot10^{-6}$	$4.26\cdot10^{-5}$	$2.44\cdot10^{-6}$
gene		$1.58\cdot10^{-4}$	$1.65\cdot10^{-4}$	$1.94\cdot10^{-4}$	$1.14\cdot10^{-4}$	$2.66\cdot10^{-6}$	$4.14\cdot10^{-6}$	$5.21\cdot10^{-5}$	$3.27\cdot10^{-6}$
gbM		$3.09\cdot10^{-4}$	$3.52\cdot10^{-4}$	$3.38\cdot10^{-4}$	$2.30\cdot10^{-4}$	$5.08\cdot10^{-6}$	$9.43\cdot10^{-6}$	$4.62\cdot10^{-5}$	$8.22\cdot10^{-6}$
non-gbM		$1.12\cdot10^{-4}$	$1.06\cdot10^{-4}$	$1.50\cdot10^{-4}$	$8.05\cdot10^{-5}$	$2.02\cdot10^{-6}$	$3.04\cdot10^{-6}$	$1.71\cdot10^{-5}$	$2.96\cdot10^{-6}$
promoter		$9.36\cdot10^{-5}$	$6.06\cdot10^{-5}$	$1.04\cdot10^{-4}$	$4.71\cdot10^{-5}$	$1.87\cdot10^{-6}$	$3.01\cdot10^{-6}$	$2.47\cdot10^{-5}$	$2.25\cdot10^{-6}$
intergenic		$5.64\cdot10^{-5}$	$2.09\cdot10^{-5}$	$9.79\cdot10^{-5}$	$3.10\cdot10^{-5}$	$3.44\cdot10^{-6}$	$4.95\cdot10^{-6}$	$9.45\cdot10^{-6}$	$2.84\cdot10^{-6}$
TE	α	$4.51\cdot10^{-5}$	$1.28\cdot10^{-6}$	$2.57\cdot10^{-4}$	$9.60\cdot10^{-5}$	$2.66\cdot10^{-6}$	$8.73\cdot10^{-6}$	$2.84\cdot10^{-5}$	$8.14\cdot10^{-6}$
5'UTR		$7.67\cdot10^{-6}$	$1.07\cdot10^{-5}$	$2.06\cdot10^{-5}$	$1.55\cdot10^{-5}$	$9.40\cdot10^{-7}$	$9.60\cdot10^{-7}$	$7.28\cdot10^{-6}$	$1.25\cdot10^{-6}$
3'UTR		$1.09\cdot10^{-4}$	$1.06\cdot10^{-4}$	$1.74\cdot10^{-4}$	$6.54\cdot10^{-5}$	$2.96\cdot10^{-6}$	$2.90\cdot10^{-6}$	$8.63\cdot10^{-5}$	$4.02\cdot10^{-6}$
chromosome arm		$1.35\cdot10^{-4}$	$1.09\cdot10^{-4}$	$1.35\cdot10^{-4}$	$7.30\cdot10^{-5}$	$1.93\cdot10^{-6}$	$3.01\cdot10^{-6}$	$4.04\cdot10^{-5}$	$2.28\cdot10^{-6}$
pericentromere		$1.44\cdot10^{-4}$	$1.18\cdot10^{-4}$	$1.97\cdot10^{-4}$	$9.41\cdot10^{-5}$	$2.71\cdot10^{-6}$	$5.81\cdot10^{-6}$	$3.06\cdot10^{-5}$	$4.68\cdot10^{-6}$
centromere		$5.66\cdot10^{-5}$	$4.03\cdot10^{-5}$	$2.03\cdot10^{-4}$	$8.87\cdot10^{-5}$	$3.88\cdot10^{-6}$	$8.67\cdot10^{-6}$	$3.24\cdot10^{-5}$	$9.43\cdot10^{-6}$
genome-wide		$2.83\cdot10^{-4}$	$2.30\cdot10^{-4}$	$8.74\cdot10^{-4}$	$4.40\cdot10^{-4}$	$5.16\cdot10^{-6}$	$8.88\cdot10^{-6}$	$2.23\cdot10^{-4}$	$1.37\cdot10^{-5}$
gene		$9.50\cdot10^{-4}$	$7.89\cdot10^{-4}$	$1.48\cdot10^{-3}$	$9.28\cdot10^{-4}$	$1.60\cdot10^{-5}$	$1.99\cdot10^{-5}$	$4.03\cdot10^{-4}$	$2.66\cdot10^{-5}$
gbM		$7.36\cdot10^{-4}$	$6.95\cdot10^{-4}$	$9.31\cdot10^{-4}$	$6.89\cdot10^{-4}$	$1.21\cdot10^{-5}$	$1.87\cdot10^{-5}$	$1.28\cdot10^{-4}$	$2.47\cdot10^{-5}$
non-gbM		$1.27\cdot10^{-3}$	$9.30\cdot10^{-4}$	$2.43\cdot10^{-3}$	$1.33\cdot10^{-3}$	$2.30\cdot10^{-5}$	$2.68\cdot10^{-5}$	$2.81\cdot10^{-4}$	$4.93\cdot10^{-5}$
promoter		$5.92\cdot10^{-4}$	$3.16\cdot10^{-4}$	$1.24\cdot10^{-3}$	$5.88\cdot10^{-4}$	$1.19\cdot10^{-5}$	$1.57\cdot10^{-5}$	$2.98\cdot10^{-4}$	$2.82\cdot10^{-5}$
intergenic		$4.86\cdot10^{-5}$	$4.05\cdot10^{-5}$	$5.66\cdot10^{-4}$	$1.92\cdot10^{-4}$	$2.96\cdot10^{-6}$	$9.60\cdot10^{-6}$	$5.48\cdot10^{-5}$	$1.76\cdot10^{-5}$
TE	β	$1.01\cdot10^{-5}$	$3.20\cdot10^{-7}$	$1.95\cdot10^{-4}$	$7.63\cdot10^{-5}$	$6.00\cdot10^{-7}$	$2.19\cdot10^{-6}$	$2.15\cdot10^{-5}$	$6.48\cdot10^{-6}$
5'UTR		$4.51\cdot10^{-4}$	$4.82\cdot10^{-4}$	$1.65\cdot10^{-3}$	$1.29\cdot10^{-3}$	$5.56\cdot10^{-5}$	$4.32\cdot10^{-5}$	$5.91\cdot10^{-4}$	$1.04\cdot10^{-4}$
3'UTR		$1.56\cdot10^{-3}$	$1.37\cdot10^{-3}$	$4.73\cdot10^{-3}$	$1.86\cdot10^{-3}$	$4.24\cdot10^{-5}$	$3.76\cdot10^{-5}$	$2.43\cdot10^{-3}$	$1.16\cdot10^{-4}$
chromosome arm		$8.86\cdot10^{-4}$	$6.43\cdot10^{-4}$	$1.51\cdot10^{-3}$	$8.75\cdot10^{-4}$	$1.26\cdot10^{-5}$	$1.79\cdot10^{-5}$	$4.58\cdot10^{-4}$	$2.74\cdot10^{-5}$
pericenrtomere		$2.62\cdot10^{-4}$	$1.99\cdot10^{-4}$	$7.82\cdot10^{-4}$	$3.92\cdot10^{-4}$	$4.94\cdot10^{-6}$	$9.77\cdot10^{-6}$	$1.22\cdot10^{-4}$	$1.95\cdot10^{-5}$
centromere		$1.25\cdot10^{-5}$	$8.32\cdot10^{-6}$	$9.41\cdot10^{-5}$	$4.25\cdot10^{-5}$	$8.60\cdot10^{-7}$	$1.79\cdot10^{-6}$	$1.50\cdot10^{-5}$	$4.52\cdot10^{-6}$
genome-wide		2.19	2.18	5.28	5.61	0	0	0	0
gene		5.99	4.79	7.63	8.1	0	0	0	0
gbM		2.38	1.98	2.75	2.99	0	0	0	0
non-gbM		11.32	8.78	16.21	16.55	0	0	0	0
promoter		6.32	5.21	12	12.48	0	0	0	0
intergenic		0.86	1.94	5.78	6.19	0	0	0	0
TE	β/α	0.22	0.25	0.76	0.79	0	0	0	0
5'UTR		58.83	44.95	80.11	82.95	0	0	0	0
3'UTR		14.24	12.9	27.18	28.49	0	0	0	0
chromosome arm		6.54	5.92	11.22	11.99	0	0	0	0
pericenrtomere		1.82	1.68	3.97	4.17	0	0	0	0
centromere		0.22	0.21	0.46	0.48	0	0	0	0

Table S2: Epimutation rates estimates for regions genome-wide and regions overlapping with different annotations. mCG gain rate (α), mCG loss rate (β) and β/α ratio per MA line. The last 4 column detail the standard error (SE) associated with each rate. These are calculated from bootstrapping.
